# Potential of Imidazolium-Based Ionic Liquids for Sustainable Enhancement of Oil Recovery in Emirati Tight Reservoirs

**DOI:** 10.1021/acsomega.5c03875

**Published:** 2025-07-16

**Authors:** Noran Mousa, Basim Abu-Jdayil, Abdulrazag Y. Zekri

**Affiliations:** † Chemical & Petroleum Engineering Department, 11239 United Arab Emirates University, P.O. Box 15551, Al Ain, United Arab Emirates

## Abstract

In response to the increasing global demand for energy and the limitations of conventional oil recovery methods, this study investigates the potential of imidazolium-based ionic liquids (ILs) as novel agents for enhanced oil recovery (EOR). The effects of four imidazolium ILs—1-decyl-3-methylimidazolium chloride, 1-dodecyl-3-methylimidazolium chloride, 1-dodecyl-3-methylimidazolium tetrafluoroborate, and 1-hexadecyl-3-methyl imidazolium bromide (C_16_mimBr)—on phase behavior, interfacial tension (IFT), and wettability between oil, water and rock were examined in the context of Emirati tight oil reservoirs. In addition, the thermal stability of these ILs was investigated under extreme temperatures of up to 600 °C using thermogravimetric analysis. Experiments were conducted across a range of temperatures (up to 110 °C), IL concentrations (0, 500, 1500, and 3000 ppm), and salinities (seawater and formation brine). In particular, the IL with the longer alkyl chain (C_16_mimBr) effectively reduced the IFT by >99% in formation brine at 3000 ppm and 110 °C and altered contact angles to as low as 15.37°. Emulsification experiments were conducted with the emulsions analyzed under a microscope to measure the size of water droplets within the microemulsions. It was observed that all imidazolium ILs tested at 500 ppm successfully stabilized oil/brine emulsions, maintaining complete phase integrity for at least 48 h. Imidazolium ILs with longer chains and bulky anions (C_12_mimBF_4_) showed adsorption as low as 2.71 mg/g, reducing chemical loss and enhancing EOR effectiveness. The findings indicate that the choice of IL and its concentration significantly impact the economic reduction of IFT and optimize EOR processes.

## Graphical abstract

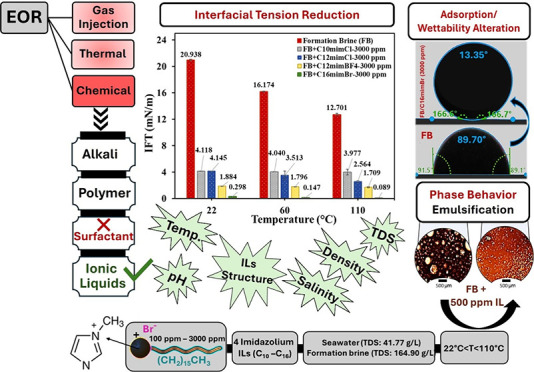

## 1 Introduction

The rising energy demand requires the development of novel techniques for enhanced oil recovery (EOR). In 2017, the Energy Information Administration estimated a 28% rise in global energy consumption between 2015 and 2040, highlighting the expected growth in demand for various energy sources, including renewable energy sources alongside traditional fossil fuels.^1^ Considerable amounts of petroleum resources remain unrecoverable using conventional techniques. After primary and secondary flooding, approximately 45–55% of the original oil in place may be extracted.^2^ EOR technologies are employed to recover residual oil, addressing the increasing global energy demand. Many traditional surfactants are not biodegradable, pose risks to ecosystems and are instable. Traditional surfactant flooding becomes inefficient due to instability in harsh environments, leading to reduced oil recovery.^3^ Another significant problems is the adsorption of traditional surfactants onto reservoir rocks, which leads to a substantial loss of the active agent and increased chemical consumption.^4^

Recently, ionic liquids (ILs) have been shown to exhibit promising performance in lowering residual oil saturation.^5^ ILs are compounds comprising solely of ions and have melting points below 100 °C.^6^ They have distinctive chemical and physical properties, making them effective substitutes for organic solvents with a volatile nature. These compounds exhibit very low vapor pressure and excellent chemical and thermal stability. In addition, they can dissolve a broad variety of polar, nonpolar, organic, and inorganic molecules.^6^ Imidazole-based ILs offer various advantages, such as relatively low cost, low toxicity, biodegradability, and water solubility.^7,8^ Furthermore, ILs may break down asphaltene aggregates, allowing the release of light hydrocarbons and improving the flow ability of crude oil.^9^ Organic cations include asymmetric N-cyclic molecules, such as imidazolium derivatives, whereas anions include halides, tetrafluoroborate, methanesulfonate, hexafluorophosphate, and more complicated organic anions. These anions are appropriate for high temperature and pressure conditions and have variable degrees of hydrophobicity.^10^

IFT reduction is a key mechanism for EOR, facilitating the mobilization of trapped oil, enhancing microemulsion formation, and improving the efficiency of oil recovery. The studies indicate that longer-chain imidazolium ILs reduce IFT more effectively than shorter-chain ILs. In southern Iran, Barari et al. (2022) observed a significant IFT reduction from 10.59 to 2.06 mN/m using (C_18_mimCl) at 1000 ppm.^11^ Similarly, Tackie-Otoo et al. (2022) recorded an IFT of 0.055 mN/m with (C_16_mimBr) in Malaysian oil fields, which was further reduced to 0.0076 mN/m with the addition of ETA.^12^ Also, they observed a significant reduction in contact angle from 116° to 60° using (C_16_mimBr) at a low concentration of 300 ppm, and higher IL concentrations can further improve wettability change.^12^ In general, long alkyl chain ILs have demonstrated notable efficiency in modifying interfacial properties. For example, using 500 ppm of (C_18_mimBr) significantly reduced interfacial tension (IFT) to 0.56 mN/m and shifted the wettability of carbonate surfaces from strongly oil-wet (∼145°) to water-wet (55.5°) under high salinity conditions.^13^

On the other hand, Esfandiarian et al. (2021) found that shorter-chain ILs like (C_6_mimCl) and (C_8_mimCl) had higher IFT values even at high concentrations, such as 6.35 mN/m for (C_6_mimCl) at 5000 ppm.^14^ Scientists generally agree that ILs with longer alkyl chains are more efficient at lowering IFT and altering wettability, as they create stronger hydrophobic interactions at the oil–water interface. Moreover, the temperature also accelerates IFT reduction through enhanced molecular interactions. At 80 °C, (C_12_mimCl) reduced IFT to as low as 3.54 mN/m in diluted seawater at 5000 ppm.^14^ This suggests that higher temperatures enhance IL diffusion and interaction at the interface.

Different salts impact IFT reduction depending on their ionic composition. Barari et al. (2021) observed more pronounced reductions by varying salt ions and their concentration, with (C_12_mimCl) at 500 ppm achieving IFT values of 0.54 and 1.37 mN/m in NaCl (40,880 ppm) and Na_2_SO_4_ (33,086 ppm) solutions, respectively at 25 °C.^15^ This indicates that sulfate ions may interfere with IL performance due to stronger ion pairing with IL cations. In contrast, Nandwani et al. (2018) observed that high-salinity NaCl brine (350,000 ppm) did not inhibit the performance of (C_16_mimBr) at 6000 ppm, which reduced IFT from 11.5 to 0.12 mN/m.^16^

Selecting effective surfactants for enhanced oil recovery requires thorough evaluation of various factors, including reservoir temperature, salinity, formation characteristics, and pH.^17^ Additionally, the rock’s permeability, the surfactant’s molecular structure and cost, its adsorption behavior on the reservoir matrix, and its resulting impact on oil recovery must all be considered to ensure compatibility with specific reservoir conditions.^18^ However, in enhanced oil recovery processes, the interaction between injected surfactants and reservoir rocks can result in significant adsorption losses.^19–21^ This phenomenon reduces the availability of active surfactants in the pore spaces, limiting its effectiveness in altering interfacial tension and wettability. Consequently, evaluating the adsorption behavior of surfactants and alternative agents such as ionic liquids is crucial for optimizing performance and ensuring economic viability.^22^

While these studies have laid a strong foundation for using imidazolium ionic liquids in EOR, a gap exists in the comprehensive exploration of the optimal conditions for their use. Imidazolium-based ILs have not been used in Emirati oil reservoirs, especially tight ones. This research utilizes four imidazolium-based ILs: 1-decyl-3-methylimidazolium chloride (C_10_mimCl), 1-dodecyl-3-methylimidazolium chloride (C_12_mimCl), 1-dodecyl-3-methylimidazolium tetrafluoroborate (C_12_mimBF_4_), and 1-hexadecyl-3-methylimidazolium bromide (C_16_mimBr), along with Emirati crude oil. It provides a direct comparison of structure–function relationships among ILs with varying alkyl chain lengths and anions (Cl^–^ vs BF_4_^–^) and provides unique insights into the molecular design for EOR. Also, evaluating the thermal degradation behavior of ILs for EOR under such high temperatures using TGA is not commonly addressed and adds value for field deployment. The phase behavior, microemulsion type, emulsion stability over time, adsorption, wettability (contact angle) using the sessile drop method, and IFT using the spinning drop method under a wide range of salinities and temperatures (up to 110 °C) were investigated.

This study postulates that the physical and chemical characteristics of ILs strongly influence the success of EOR techniques. The primary objective of this research was to examine the impacts of these properties on the efficiency of ILs in altering the wettability of oil and reducing the IFT between oil and IL solutions. Furthermore, the most favorable water salinities to dilute certain ILs were determined. The synergetic effects and relations between IL concentration, temperature, and salinity were investigated. In addition, the observed dramatic wettability alteration in tight rocks using ILs is a key novelty that impacts oil displacement efficiency. Also, using microscopy to analyze emulsion microstructure and droplet size provides a molecular-level understanding of IL performance, which is not widely reported in EOR literature. For an in-depth analysis, factors such as ionic strength, anion type, density of IL solution, total dissolved solids (TDSs), conductivity, and pH were considered to extend the scope of discussion and analysis beyond previous research efforts.

## 2 Experimental Section

### 2.1 Materials

In this research, SA oil was provided by Abu Dhabi National Oil Company (ADNOC), Abu Dhabi, United Arab Emirates (UAE). Table 1 presents the physical properties of SA light crude oil, including water content, density, molecular weight, API°, and SARA analysis.

**Table 1:** Physical and Compositional Properties of the Used Crude Oil

Well Name	SA Field
Free Water (cm^3^)	0
Water Content (wt %)	<0.1
Density at STP (g/cm^3^)	0.8354
API°	37.71
Molecular Weight (g/mol)	201.0
Saturates (%)	41.5
Aromatics (%)	30.5
Resins (%)	24.6
Asphaltenes (%)	3.5

The current study utilized C_10_mimCl, C_12_mimCl, C_12_mimBF_4_, and C_16_mimBr (Ambeed, USA). Their chemical structures, drawn using MolView, are presented in Scheme 1. They feature a methyl-substituted imidazolium ring with varying alkyl chains (C_10_–C_16_) and different anions (Cl^–^, BF_4_^–^, Br^–^).

**Scheme 1:**
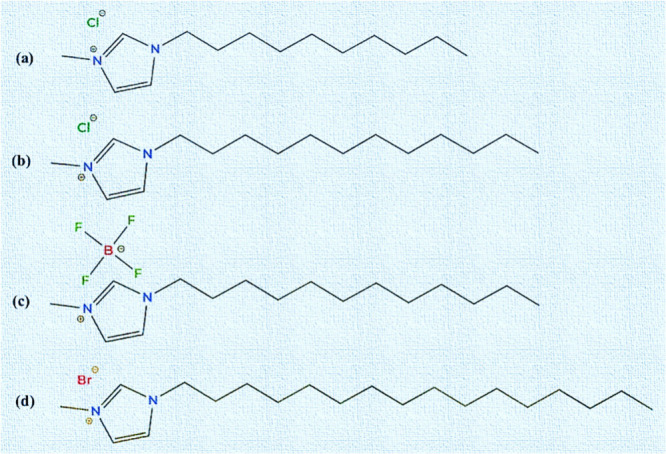
Chemical Structures of the Imidazolium-Based Ionic Liquids Used in This Study: (a) C_10_mimCl, (b) C_12_mimCl, (c) C_12_mimBF_4_, and (d) C_16_mimBr

All ILs were diluted in the prepared seawater (TDS: 41.77 g/L) and formation brine (TDS: 164.90 g/L) at 100, 500, 1500, and 3000 ppm. The salt compositions of the seawater and formation brine solutions are presented in Table 2.

**Table 2:** Salts Used in the Preparation of Seawater and Formation Brine

Salt Name	Composition of Synthetic Seawater Salts (g/L)	Composition of Synthetic Formation Brine (g/L)
NaCl	29.80	157.08
CaCl_2_·2H_2_O	1.86	60.28
MgCl_2_·6H_2_O	13.53	14.23
Na_2_SO_4_	5.00	0.39
NaHCO_3_	0.23	0.17
KCl	0.92	—

### 2.2 Phase Behavior and Microscopic Analysis

In the first stage, the interaction compatibility of the ILs dissolved in different types of water was evaluated. To do this, we prepared various solutions of the ILs at 500, 1500, 3000, and 5000 ppm using seawater and formation brine. Consequently, we let these solutions stand for several days and observe any indication of solid formation, aiming to identify the concentrations of chemicals that result in a clear IL solution with no precipitation in both seawater and formation brine. Therefore, the IL with a concentration of 5000 ppm was excluded.

To gather essential data, emulsification experiments were performed using 10 mL vials.^23^ A uniform 1:1 volumetric proportion of SA oil and an IL solution (500 ppm), diluted in seawater or formation brine, was mixed by gently agitating the vials by shaking them in a right and left manner for 5 min. Subsequently, the emulsion underwent a 24-h incubation period at 90 °C. The examination of these emulsions was conducted using Olympus stereomicroscopes (SZ2-ST, Japan), and the average size of the dispersed water droplets within the microemulsions was quantitatively analyzed utilizing the ImageJ software.

### 2.3 Emulsion Stability Evaluation

High stability water-in-oil emulsions were prepared by mixing 50 vol % SA crude oil with 50 vol % of IL solution (500 ppm), previously diluted in either seawater (SW) or formation brine (FB). The mixture was blended in a beaker using a magnetic stirrer at 1500 rpm for 1 h to ensure uniform emulsification. Following mixing, the emulsions were transferred to graduated vials and allowed to stand under static conditions at ambient temperature. Emulsion stability was assessed by monitoring the volume of separated water over time. The demulsification efficiency (DE%) was calculated using the following equation:

1 $$\mathrm{DE}\%=\frac{\left(\mathrm{Water separated}\left(\mathrm{mL}\right)\mathrm{with respect to time}\right)}{\left(\mathrm{Initial total water}\left(\mathrm{mL}\right)\mathrm{in emulsion}\right)}\times 100$$

### 2.4 TGA

TGA was used to determine the degradation temperatures of C_10_mimCl, C_12_mimCl, C_12_mimBF_4_, and C_16_mimBr using a thermogravimetric analyzer (Q-50, TA Instruments, New Castle, DE, USA). Under a nitrogen atmosphere (100 mL/min), samples (10–15 mg) were heated from 20 to 600 °C at a heating rate of 10 °C/min. The thermal stability of the samples was calculated according to the ASTM E2550 method.

### 2.5 Physical Characterization

The physical properties (pH, TDS, and conductivity) of the four ILs diluted in seawater and formation brine at 100, 500, 1500, and 3000 ppm were measured using a multiparameter meter (ULTRAMETER II, MYRON-6PFC, Carlsbad, California, USA) and recorded in Tables 3 and 4. In addition, the densities of the IL solutions were measured using a pycnometer according to ASTM D854 using the following equation:

2 $$\mathrm{Density}=\frac{\left(\mathrm{Mass of filled pycnometer}-\mathrm{Mass of empty pycnometer}\right)}{\left(\mathrm{Volume of the pycnometer}\right)}$$

**Table 3:** Physical Properties of the Prepared IL Solutions in Seawater Solution

Concentration (ppm)	pH	TDS (PPT)	Conductivity (mS)	Density (g/cm^3^)
Seawater (SW)				
0	7.85	41.77	62.42	1.037
C_10_mimCl_SW				
100	7.72	41.87	62.47	1.037
500	7.81	41.94	62.6	1.037
1500	7.57	41.56	62.06	1.026
3000	7.77	42.15	62.85	1.016
C_12_mimCl_SW				
100	7.71	40.96	61.53	1.037
500	7.61	42.63	63.43	1.037
1500	7.7	41.95	62.58	1.031
3000	7.68	42.47	63.24	1.025
C_12_mimBF_4__SW				
100	7.43	42.12	62.73	1.037
500	6.3	41.43	61.94	1.037
1500	2.69	41.98	62.93	1.037
3000	2.52	42.85	63.62	1.037
C_16_mimBr_SW				
100	7.68	41.97	62.65	1.037
500	7.37	42.08	62.76	1.036
1500	7.89	39.56	59.71	1.021
3000	7.69	41.74	62.35	1.006

**Table 4:** Physical Properties of the Prepared IL Solutions in Formation Brine

Concentration (ppm)	pH	TDS (PPT)	Conductivity (mS)	Density (g/cm^3^)
Formation Brine (FB)				
0	6.28	164.9	200.5	1.14
C_10_mimCl_FB				
100	6.62	165.3	200.4	1.124
500	6.69	161.5	196	1.129
1500	6.55	166.4	201.6	1.136
3000	6.57	170.2	206	1.145
C_12_mimCl_FB				
100	6.39	174.3	210.9	1.122
500	7.07	173.4	219.9	1.126
1500	6.93	166.4	201.6	1.135
3000	6.75	169.1	204.9	1.143
C_12_mimBF_4__FB				
100	6.5	173.6	210	1.123
500	2.42	160.7	195.2	1.126
1500	1.47	168.3	204.6	1.133
3000	1.33	170.7	206.6	1.142
C_16_mimBr_FB				
100	5.73	173.9	210.5	1.119
500	6.27	164.3	198.7	1.123
1500	6.44	168.2	203.7	1.137
3000	6.38	170.4	206.5	1.143

### 2.6 IFT Testing

The IFT of the four diluted ILs in seawater and formation brine at 100, 500, 1500, and 3000 ppm and 25, 60, and 110 °C was measured in contact with SA oil using a spinning drop tensiometer (SDT, Kruss, Germany). The spinning drop method was used to determine the IFT of two nonmixable liquids using the SDT. A capillary with an inner diameter of 3.25 mm was prepared prior to testing via a heavy phase (IL/brine) and a lighter phase (crude oil droplet) (Figure 1a). The IFT is proportional to the diameter or curvature of the drop, which is pulled lengthwise owing to centrifugal force. Image analysis was performed using the Young–Laplace evaluation technique via the Advance program. In the Image analysis tile, the Advance software shows an accurate drop image for each measurement (Figure 1b).

**Figure 1:**
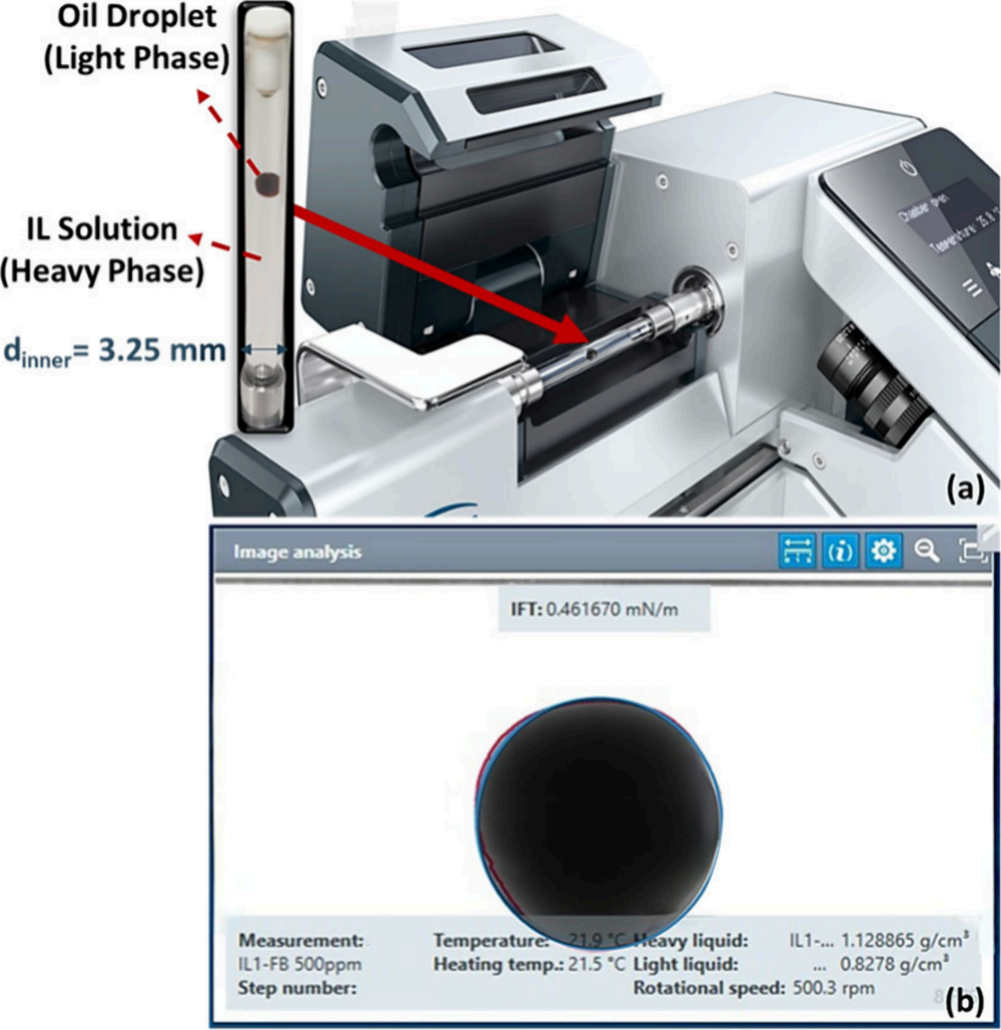
Interfacial tension testing: a) experiment setup and b) software workspace of IL solution and crude oil.

The critical micelle concentration (CMC) was determined using the two-line intersection method, a widely accepted approach for analyzing interfacial tension (IFT) versus concentration data. In this method, two linear regressions are applied: one to the steeply decreasing region before micelle formation and another to the plateau region where further IL addition has minimal effect on IFT. The CMC is identified as the intersection point of these two linear segments, representing the concentration at which micelles begin to form in the bulk phase.^24^

### 2.7 Core Sample Preparation and Characterization

Core samples were trimmed using a high-speed shredder and then cleaned with toluene and methanol using Soxhlet extraction. After cleaning, the samples were dried, and weight and dimensions were measured using a balance and digital Vernier caliper, respectively. Finally, the porosity and permeability of the core samples were tested under N_2_ gas using Poroperm from Vinci technologies.

X-ray powder diffraction measurements were conducted for the chosen core samples using an X’pert-3-Powder X-ray diffractometer (Analytical, Philips, Netherlands) with Cu Kα radiation and a diffracted-beam monochromator. A metallic sample holder with a diameter of 16 mm and a depth of 1 mm was filled with fine powder, which was consequently pressed and smoothed with flat glass. A loaded sample weight between 0.4 and 0.5 g was used to ensure proper sample compactness. Diffraction patterns were recorded in the 2θ range of 10°–90° with a step increment of 0.030°, and each step was counted for 1 s. The total scan time for each sample was 15 min, with the generator set at 45 kV and 40 mA. The Emirati core samples showed calcite (limestone carbonate) cores (Figure 2).

**Figure 2:**
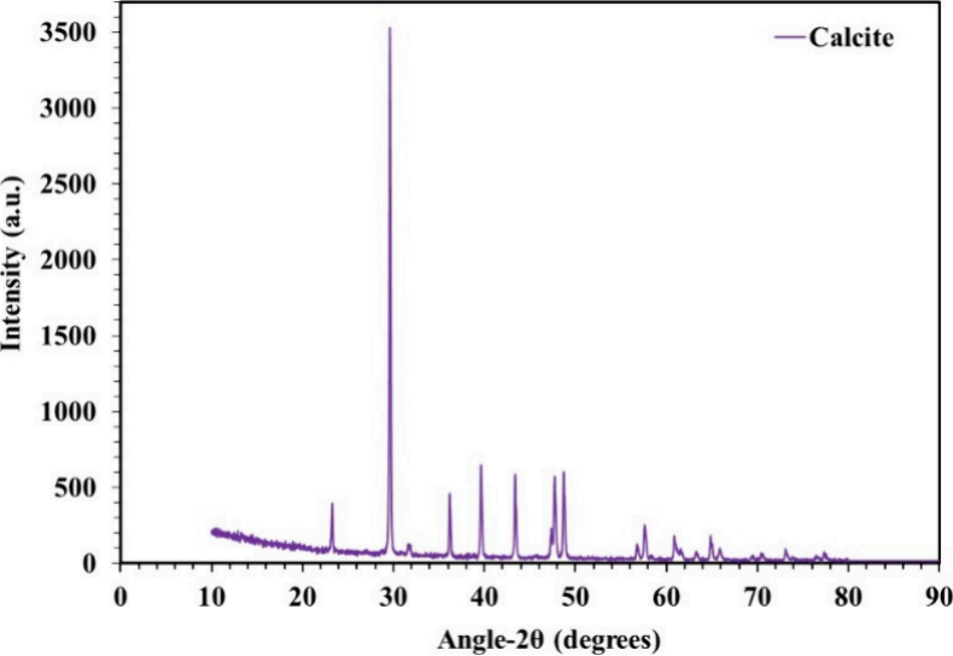
X-ray diffraction examination of the carbonate core specimen.

### 2.8 Wettability (Contact Angle Tests)

To assess the effects of the four ILs at 500 and 3000 ppm on the alteration of wettability in carbonate thin sections, we measured the contact angles formed by an oil droplet on various aged carbonate surfaces immersed in both seawater and formation brine using the drop shape analyzer (DSA25 Basic, Kruss, Germany). We employed the sessile drop method, which involved placing an oil droplet beneath the carbonate thin section with a needle diameter of 0.5 mm, which had been immersed in the IL solution, within a glass cuvette (SC02) with inner dimensions of 36 mm × 36 mm × 30 mm (W × D × H) (Figure 3). High-resolution images of oil droplets were captured using a CF04 camera, and contact angles were subsequently determined using the Advance DropShape software. To ensure accuracy and reliability, each experiment was conducted three times to reduce potential experimental errors and verify repeatability. All measurements were consistently performed at 60 °C.

**Figure 3:**
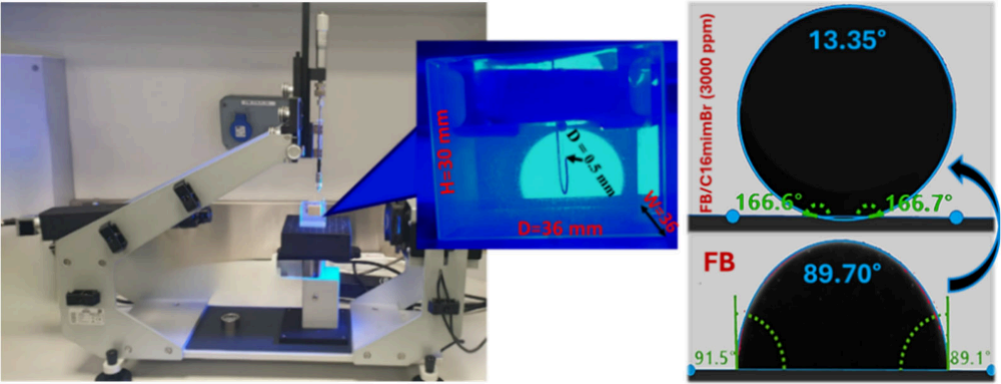
Wettability (contact angle) measurement setup.

### 2.9 Adsorption Analysis

The adsorption of ionic liquids (ILs) onto carbonate rock was assessed using a batch equilibrium approach. For each experiment, 1 g of finely crushed carbonate rock was added to 25 mL of IL solution, initially prepared at a concentration of 3000 ppm and diluted in formation brine. The mixtures were agitated using a horizontal shaker for 24 h at ambient temperature to reach adsorption equilibrium. Afterward, the suspensions were centrifuged to separate the rock from the supernatant.

The remaining IL concentration in the aqueous phase was determined using a UV–vis spectrophotometer (SPECTROstar Nano, BMG LABTECH, Germany) in cuvette mode at 220 nm. A calibration curve was generated by measuring the absorbance of IL standard solutions at 100, 500, 1500, and 3000 ppm. Formation brine was used as the blank. The adsorption capacity (mg/g) was calculated from the difference between the initial and final IL concentrations in the solution.^25^ The adsorption density (*q*, in mg/g) was determined using the mass balance approach shown in Equation 3

3 $$q=\frac{\left(C_{0}-C_{e}\right)\times v}{m}$$

where *C*_0_ and *C*_e_ represent the initial and equilibrium IL concentrations in the solution (ppm), *v* is the volume of the IL solution (mL), and *m* is the mass of the carbonate rock sample (g). The amount of IL adsorbed onto the rock surface was calculated by comparing the IL concentration before and after equilibrium, providing the basis for quantifying static adsorption.

## 3 Results and Discussion

### 3.1 Thermal Stability

TGA was performed using a TA Instruments Q-50 thermogravimetric analyzer to determine the thermal stability and degradation temperatures of the ILs under extreme reservoir temperature conditions. The temperature of crude oil reservoirs in most fields within the UAE is approximately 120 °C (248 °F), identified as the Golden Zone in the context of temperature distribution for oil and gas reservoirs.^26^ The findings revealed degradation temperatures of 273.59, 281.18, 304.65, and 446.99 °C, accompanied by respective maximum weight losses of 30.66%, 23.16%, 17.79%, and 20.82% for C_10_mimCl, C_12_mimCl, C_16_mimBr, and C_12_mimBF_4_, respectively (Figure 4).

**Figure 4:**
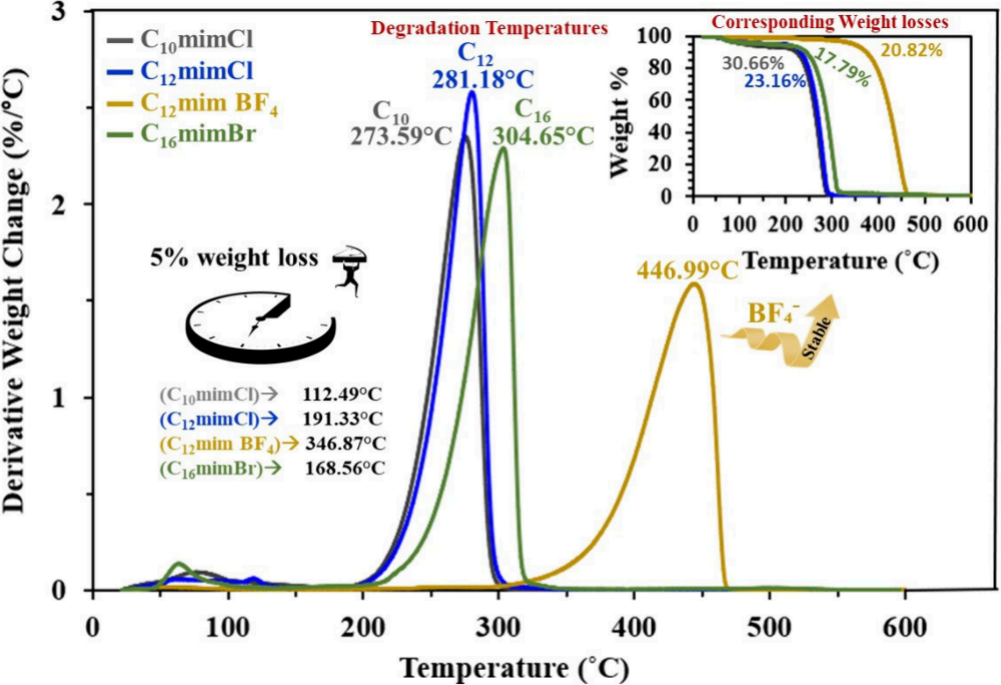
Thermogravimetric analysis of the used ILs.

The relative thermal stabilities as the weight percentages before the onset of degradation were 99%, 96%, 94%, and 93% for C_12_mimBF_4_, C_16_mimBr, C_12_mimCl, and C_10_mimCl, respectively. Figure 4 shows that the ILs with longer alkyl chains such as C_12_mimBF_4_ and C_16_mimBr demonstrated superior stability under high reservoir temperatures, indicating that longer alkyl chains contribute to enhanced stability performance. Moreover, C_12_mimBF_4_ exhibited relatively higher thermal stability compared to C_16_mimBr owing to the superior thermal stability of BF_4_ anions compared with that of Cl or Br anions.^27^

### 3.2 Phase Analysis and Microscopy

Understanding the phase behavior of aqueous IL solutions with crude oil is essential for the optimization of chemical flooding processes. We investigated the phase behavior between light crude oil and four imidazolium-based ILs diluted in seawater and formation brine at 500 ppm and 90 °C after 24 h. The oil–water interface after demulsification was examined using Olympus stereomicroscopes, and the diameters of dispersed water droplets in oil were analyzed using the ImageJ software. The microemulsions were classified into three categories based on Winsor microemulsion classification: Type I was identified by an oil-in-water phase; Type II was distinguished by a water-in-oil phase; and Type III was characterized by a bicontinuous intermediate phase comprising oil and water in equal proportions.^28^

All the prepared water-in-oil emulsions using the imidazole-based ILs exhibited Winsor Type III microemulsions, which are favorable for EOR. In Winsor Type III systems, a decrease in the middle-phase volume occurred as the temperature rose from 25 to 90 °C in the emulsions owing to the IL’s solubility in both oil and water phases. The magnification of the interface between oil and seawater in the presence of the ILs is depicted in Figure 5. The observed texture exhibits a sequential pattern of water droplets in continuous oil, characteristic of microemulsions falling within their typical diameter range. Longer-alkyl-chain ILs were able to break the accumulated oil more strongly than shorter-alkyl-chain ILs (Figure 5). The average water droplet diameters in the oil–seawater interface were 109.9, 84.68, 59.75, 55.952, and 27.78 μm for no added IL, C_10_mimCl, C_12_mimBF_4_, C_12_mimCl, and C_16_mimBr, respectively.

**Figure 5:**
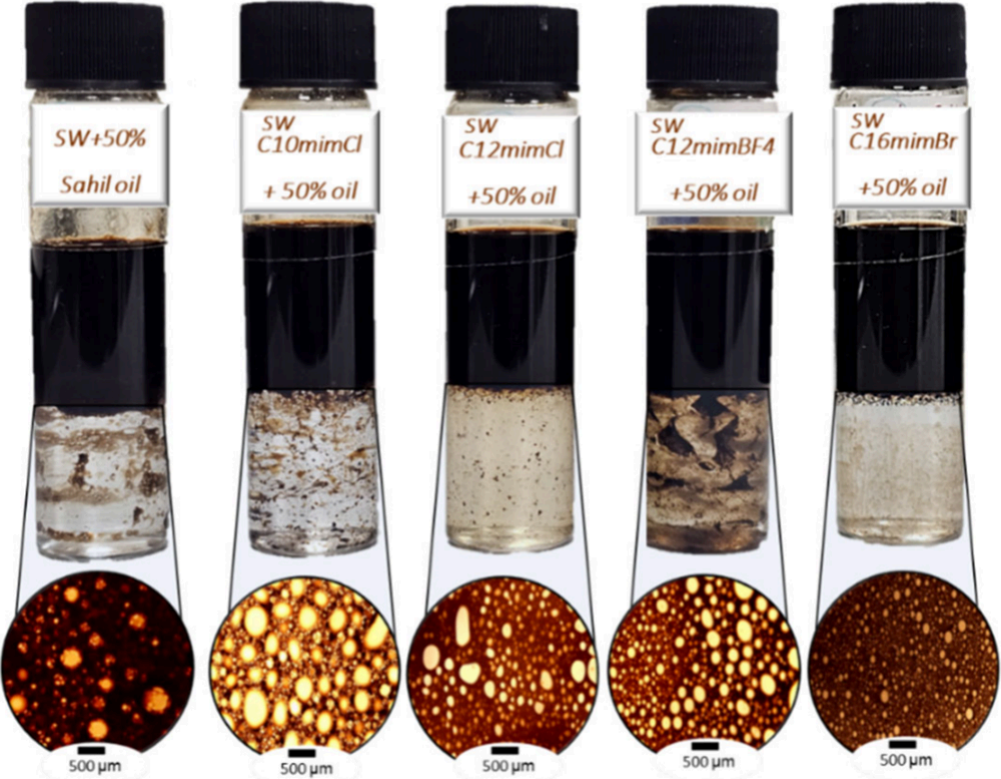
Phase behavior and microscopic analysis of seawater–oil emulsions using four ionic liquids at 90 °C.

Switching from seawater to formation brine substantially alters the phase behavior owing to changes in brine salinity (Figure 6). The yellowish-brown shades of the water phase in the presence of C_10_mimCl, C_12_mimCl, and C_12_mimBF_4_ indicate the existence of dissolved organic substances in higher salinity water/IL, such as hydrocarbons or other elements originating from the oil phase, which may have influenced coloration by dissolving into the water.^29^ The mean diameters of water droplets at the interface of oil and formation brine are as follows: 84.61 μm for no added IL, 81.81 μm for C_10_mimCl, 77.78 μm for C_12_mimBF_4_, 62.9 μm for C_12_mimCl, and 17.5 μm for C_16_mimBr. The tube test results indicate that systematically altering salinity results in reduced IFTs and increased oil and water solubilization, forming a relatively higher volume middle-phase microemulsion, consistent with previous findings.^30,31^

**Figure 6:**
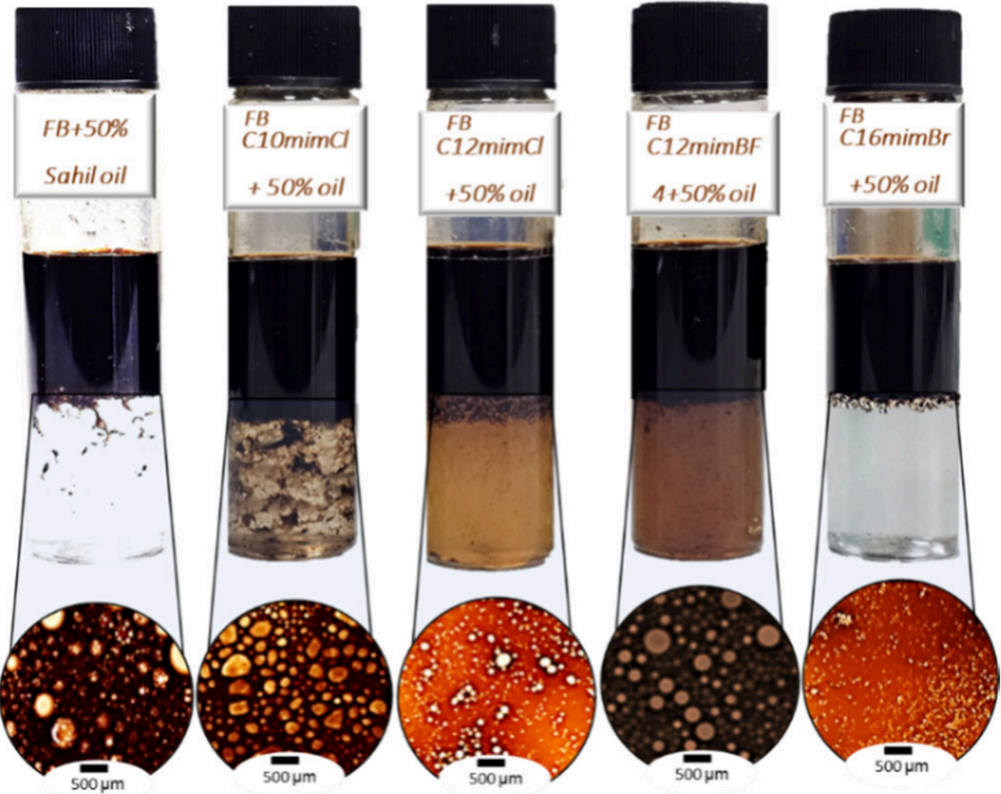
Phase behavior and microscopic analysis of formation brine–oil emulsions using four ionic liquids at 90 °C.

### 3.3 Emulsion Stability over Time

Emulsion stability plays a vital role in enhanced oil recovery (EOR), as stable emulsions can improve sweep efficiency, and enhance oil displacement in porous media. Figure 7 presents the demulsification efficiency (DE%) over time for emulsions stabilized with ILs at a fixed concentration of 500 ppm, using both SW and FB. As shown, the blank emulsions (SW/Oil and FB/Oil) demonstrated the fastest separation after 24 h at room temperature, achieving nearly 100% DE by 140 h, whereas the addition of ILs significantly delayed demulsification. All IL-stabilized systems showed no visible phase separation during the first 48 h. This indicates high emulsion stability in the presence of ILs under the tested conditions. Similarly, recent studies have reported that ionic liquid-based oil/water emulsions remained stable from 1 to 3 days without coalescence.^32^ This stability is mainly due to the formation of strong interfacial films that prevent droplet aggregation. Notably, emulsions containing C_16_mimBr exhibited the lowest DE values, followed by those with C_12_mimCl, indicating that increasing the alkyl chain length of the IL cation enhances emulsion stability. This observation is consistent with previous studies, which reported that longer alkyl chains increase IL adsorption and form more rigid interfacial films that resist droplet coalescence.^25,33^ Moreover, a comparison between C_12_mimCl and C_12_mimBF_4_ shows that emulsions with Cl^–^ counterions typically form more tightly bound and stable interfacial structures than those with BF_4_^–^.^34^ Additionally, the emulsions prepared in formation brine (Figure 7b) were slightly more stable than those in seawater (Figure 7a), which can be attributed to the higher ionic strength and divalent ion content in FB that compress the electrical double layer and promote tighter IL packing at the interface.^35^ Overall, the results confirm that all tested ILs at 500 ppm act as effective stabilizers, and their performance is strongly influenced by both the IL structure and the composition of the brine phase.

**Figure 7:**
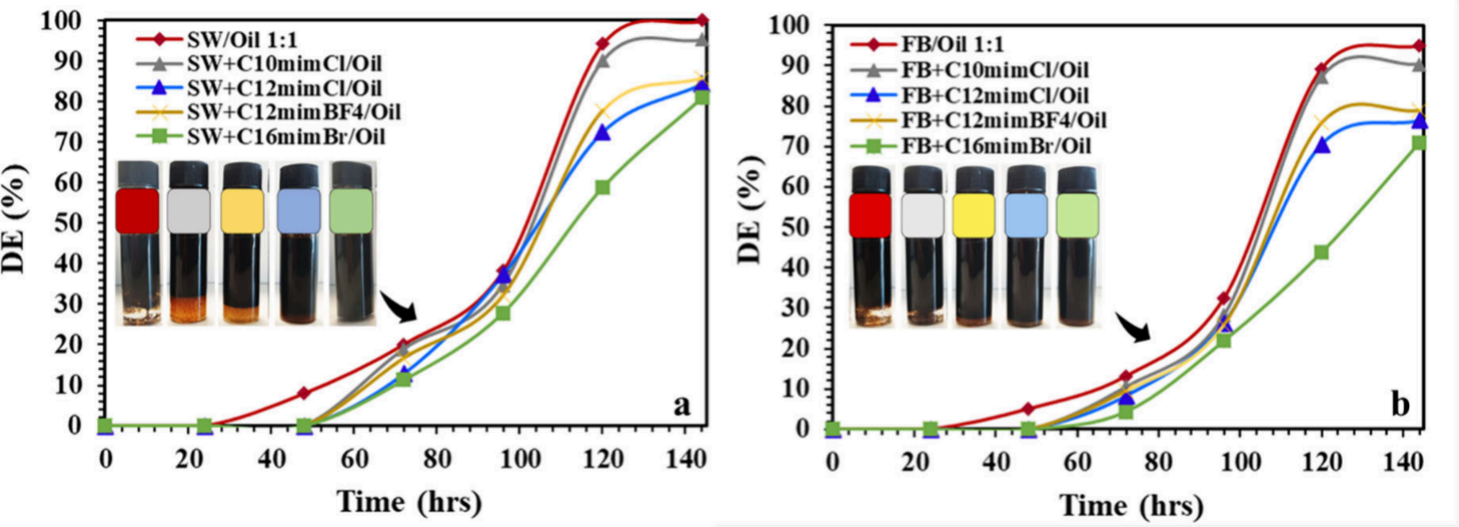
Demulsification of IL-stabilized emulsions at 500 ppm over time: (a) seawater/oil and (b) formation brine/oil systems.

### 3.4 IFT Investigation

#### 3.4.1 Effects of IL Concentration on IFT

Employing surfactants in chemical EOR techniques relies on lowering IFT and modifying wettability; hence, the surface characteristics of ILs have been explored for their potential use in EOR processes. The densities of the prepared IL solutions were measured prior to IFT testing in seawater and formation brine (Tables 3 and 4, respectively). The densities of the ILs, when diluted in seawater, slightly decreased with the rise in IL concentration up to 3000 ppm. In seawater, the dilution of ILs may decrease the density owing to the dispersion of IL ions among a relatively less dense medium (water with dissolved salts) and potential interactions with water molecules that result in volume expansion.

Conversely, in the formation brine system, the IL solution density increased only slightly at higher IL concentrations because the formation brine typically has a higher ionic strength than seawater owing to the presence of higher concentrations of salts, such as NaCl, CaCl_2_, and MgCl_2_ (Table 2). This increase in ionic strength enhances electrostatic interactions among ions, potentially forming a more compact construction and slightly increasing the density of the solution at higher IL concentrations.^36^

The impacts of varying concentrations of ILs in both seawater and formation brine on the IFT of the IL and crude oil system were assessed at a controlled room temperature of 22 °C. Figure 8 shows considerable reduction in IFT between seawater and SA oil at room temperature, which decreased from 23.56 to 9.32, 3.27, 4.29, and 2.48 mN/m, corresponding to a reduction of 60.42%, 86.08%, 81.79%, and 89.47% with the introduction of C_10_mimCl, C_12_mimBF_4_, and C_16_mimBr at a concentration of 500 ppm, respectively. Similarly, the IFT between formation brine and SA oil decreased from 20.93 to 4.45, 4.23, 4.52, and 0.35 mN/m, reflecting reductions of 78.71%, 79.75%, 78.41%, and a remarkable 98.30% following the addition of the same ILs at 500 ppm, respectively. These findings align with previous research, which indicates that IFT reduction is influenced by the increase in alkyl chain length.^37^

**Figure 8:**
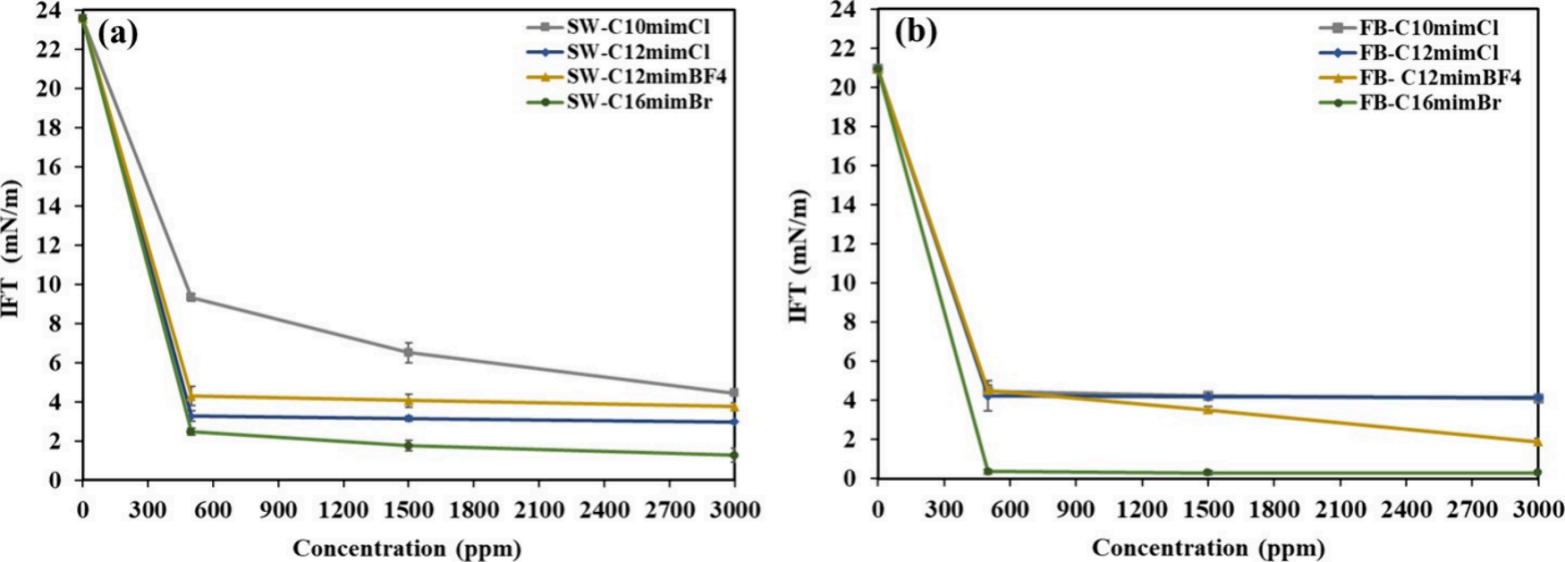
Effects of IL concentration on the IFT in (a) seawater and (b) formation brine systems.

Increasing the concentrations of C_10_mimCl, C_12_mimCl, C_12_mimBF_4_, and C_16_mimBr, from 500 to 3000 ppm in seawater considerably decreased the IFT values to 4.44, 2.97, 3.75, and 1.27 mN/m, respectively. The reduction in IFT observed when the concentrations of the ILs increased from 500 to 3000 ppm may be due to the enhanced surface activity, micellization, and the influence of hydrophobic and electrostatic interactions.^38,39^ Further examination revealed that a slightly more pronounced reduction in IFT was achieved when these ILs were diluted in formation brine at a concentration of 3000 ppm, yielding 4.11, 4.14, 1.88, and 0.29 mN/m, respectively. The presence of sulfate in seawater introduces complex ion interactions at the interface, potentially leading to a higher IFT in comparison to formation brine.^40^

The efficiency of ILs in reducing the IFT between oil and water is attributed to their amphiphilic structure, characterized by a hydrophilic imidazolium head and a hydrophobic alkyl tail. ILs are oriented near the interface between oil and water owing to this configuration. The hydrophobic tail of the ILs enters the oil layer, whereas the hydrophilic head stays in the aqueous phase. The effectiveness of this arrangement is considerably influenced by the alkyl chain’s length; longer chains strengthen hydrophobic interactions with the oil, enhancing the IL’s capacity to connect the two phases. Consequently, extended-alkyl-chain ILs are more adept at lowering the IFT as they lower the energy needed to keep the oil and water phases apart.^41^

Table 5 shows the critical micelle concentrations (CMCs) of four imidazolium-based ionic liquids in SW and FB. Moreover, a max cost-effective IFT reduction was observed around 500 ppm (CMC point) for most of the ILs. Beyond the CMC point, excess surfactant molecules form micelles instead of adsorbing at the water/oil interface, making further IFT reduction ineffective.^30^ In SW, the CMC decreases as the alkyl chain length increases.^42^ For example, C_10_mimCl has the highest CMC (1290 ppm), while C_12_mimCl, C_12_mimBF_4_, and C_16_mimBr show lower values around 500 ppm. However, FB enhances micellization and makes even shorter-chain ILs, like C_10_mimCl, effective for EOR applications. The results suggest that formation brine creates conditions favorable for micelle formation across a range of IL structures. This salt-induced leveling of CMC has been observed previously with imidazolium-based ILs, where electrolyte “salting-out” enhances micellization.^43^

**Table 5:** Critical Micelle Concentration (CMC) of Imidazolium-Based ILs in Seawater and Formation Brine

Ionic Liquid (IL)	CMC in SW (ppm)	CMC in FB (ppm)
C_10_mimCl	1290	505
C_12_mimCl	505	503
C_12_mimBF_4_	510	501
C_16_mimBr	502	500

#### 3.4.2 Effects of Temperature on IFT

The impact of temperature on the IFT of four imidazolium-based ILs was evaluated in both seawater and formation brine at 500 and 3000 ppm. Measurements were collected across a range of temperatures (22, 60, and 110 °C) to ascertain how temperature variations influence the IFT properties of these ILs in different aqueous environments. The densities of all the dilution media, IL solutions and the oil sample were measured at the desired experimental temperatures. The density showed a decreasing trend upon the temperature increase up to 110 °C as shown in Figure 9.

**Figure 9:**
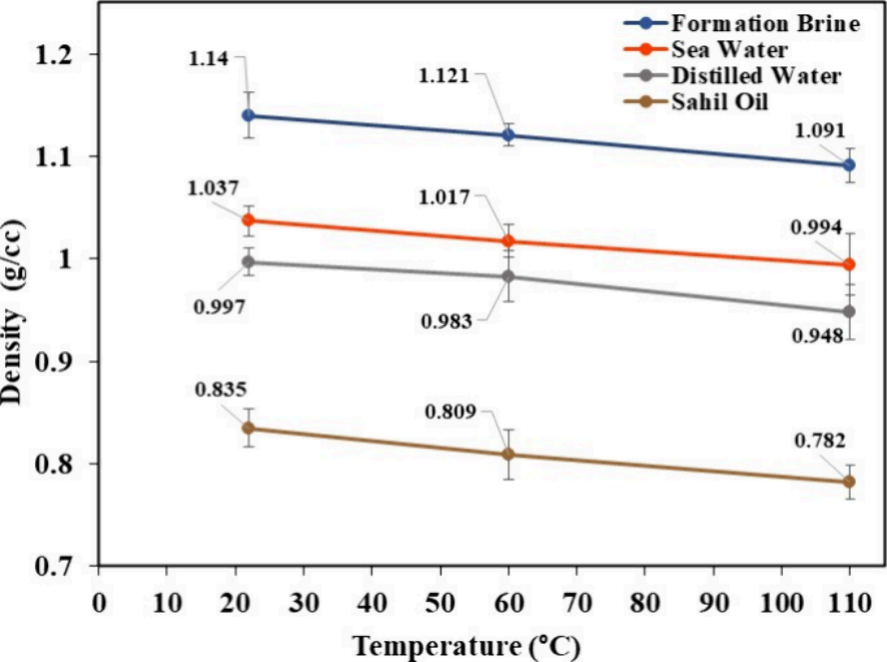
Densities of distilled water, seawater, formation brine, and light crude oil versus temperature.

Figure 10 demonstrates that as the temperature increased from 22 to 110 °C, a notable reduction was observed in the IFT between SA oil and seawater, dropping from 23.56 to 13.57 mN/m. Similarly, using formation brine, the IFT decreased from 20.94 to 12.70 mN/m, indicating a considerable impact of temperature on IFT reduction in both mediums. The rise in temperature up to 110 °C led to a noticeable decrease in the IFT of the IL–seawater solutions from 13.57 to 6.74, 2.54, 0.89, and 0.79 mN/m upon the use of C_10_mimCl, C_12_mimBF_4_, C_12_mimCl, and C_16_mimBr at 500 ppm, respectively. A similar IFT reduction trend was observed in the formation brine system (from 12.70 to 3.93, 3.83, 3.75, and 0.11 mN/m upon using the same ILs, respectively). Further reductions in IFT were obtained at 3000 ppm upon increasing the temperature from 22 to 60 and 110 °C in both seawater and formation brine (Figure 10). As the temperature increases, the thermal energy of the system also increases. This additional energy enhances the molecular motion of both oil and IL molecules, reducing the IFT. The increased molecular motion can cause molecules at the interface to rearrange more easily, reducing the energy barrier for the dispersion of one phase into another.^24^ Further, higher temperatures generally lead to decreased viscosity for both ILs and the crude oil. Reduced viscosity facilitates the mixing and movement of molecules at the interface, leading to a decrease in IFT.^44^

**Figure 10:**
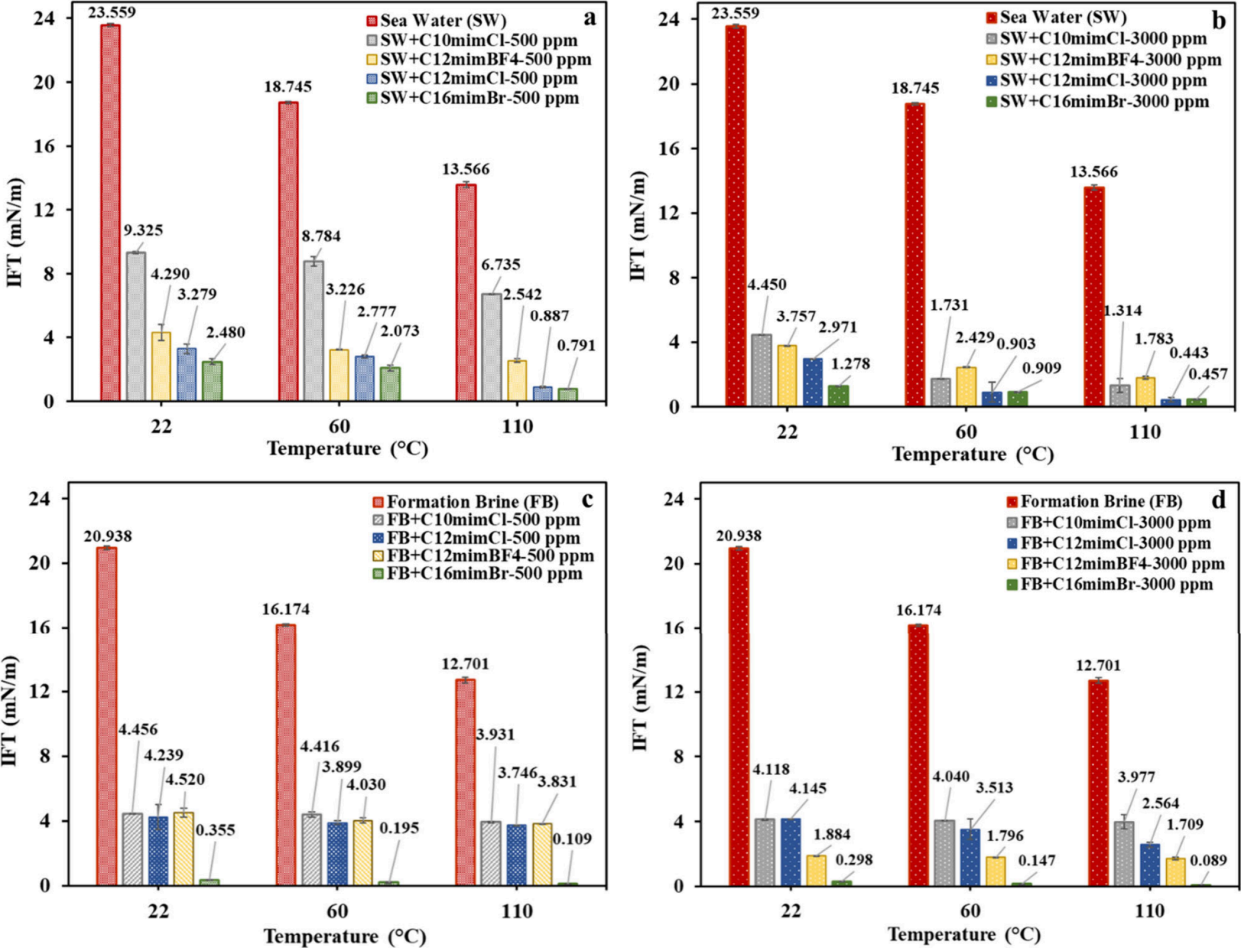
IFT measurements of SA oil and the promising ILs at 20, 60, and 110 °C in (a) seawater at 500 ppm, (b) seawater at 3000 ppm, (c) formation brine at 500 ppm, and (d) formation brine at 3000 ppm.

However, higher salt concentrations in formation brine slightly reduce the migration of shorter-alkyl-chain ILs, hindering their IFT reduction efficiency.^45^ On the other hand, the hydrophobicity of ILs increases with alkyl groups of longer carbon chains, affecting their solubility in aqueous solutions. In formation brine, the salting-out effect can enhance hydrophobic interactions among IL molecules, leading to preferential aggregation at the oil and water interface and consequently reducing the IFT more efficiently in the formation brine system.^46^ The longer-alkyl-chain IL C_16_mimBr attained the lowest IFT of 0.089 mN/m when diluted in formation brine at 3000 ppm and 110 °C (Figure 10d). In addition, divalent ions such as Mg^2+^ and Ca^2+^ in formation brine can form bridges between IL molecules and the oil phase, facilitating their adsorption at the oil–water interface.^17^ This interaction is less pronounced in seawater owing to its lower ionic strength.^47^

### 3.5 Wettability Analysis

Evaluating wettability in EOR systems to examine how oil, water, and reservoir rocks interact determines the success of the oil extraction process. According to the X-ray diffraction analysis shown in Figure 2, the reservoir core samples predominantly comprised calcite, which is cationic and attracts the anionic part of ILs, facilitating their adsorption and leading to wettability alteration.^48,49^ In the UAE, carbonate reservoirs—primarily limestone (calcite)—are the most common and dominant reservoir lithology.^50^ The isoelectric point of carbonate rock (typically between pH 8 and 9.5) plays a critical role in determining surface charge and interaction with oil or chemical agents.^51^ The porosity of the core samples was measured to be 24% ± 2.25%, whereas the permeability ranged between 4.07 and 5.84 (mD). Figure 11 shows a notable reduction in the contact angle of oil at 60 °C from 94.42° to 68.32°, 59.57°, 56.43°, and 52.83° with the incorporation of C_10_mimCl, C_12_mimCl, C_12_mimBF_4_, and C_16_mimBr at 500 ppm in seawater, whereas the contact angle in the formation brine system reduced from 90.61° to 64.48°, 61.25°, 77.83°, and 42.10°, respectively.

**Figure 11:**
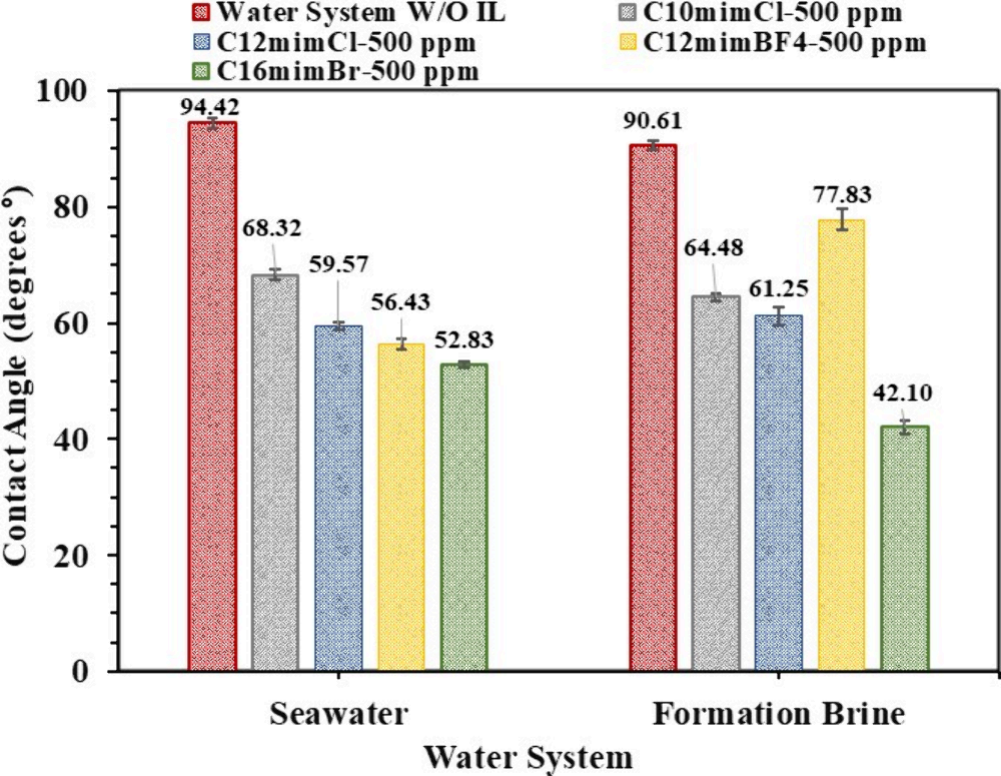
Wettability of SA oil and the promising ILs diluted in seawater and formation brine at 500 ppm.

The notable decrease in the contact angle upon the addition of imidazole-based ILs indicates that these ILs successfully modify the wettability of calcite surfaces to favor more water-wet conditions as illustrated in Scheme 2c,d. This shift toward water wettability aids in displacing oil with saline water, leading to EOR. Many studies have documented the effectiveness of ILs in changing surface wettability, demonstrating their ability to substantially reduce the contact angle on mineral surfaces and thereby improving the efficiency of oil extraction processes.^52^ The progressive reduction in contact angle observed for longer-alkyl-chain ILs (ranging from C_10_ to C_16_) indicates that the IL’s hydrophobic nature substantially influences wettability. Previous studies have emphasized the connection between the length of alkyl chains and the modification of wettability. ILs with longer alkyl chains were shown to be more efficient in chemical EOR techniques.^46^ Moreover, the notable reduction in contact angle at 60 °C can be attributed to the increase in the mobility and reactivity of IL molecules, improving their ability to absorb on the surface of the rock and shift their wettability to water wet regimes.^53^

**Scheme 2:**
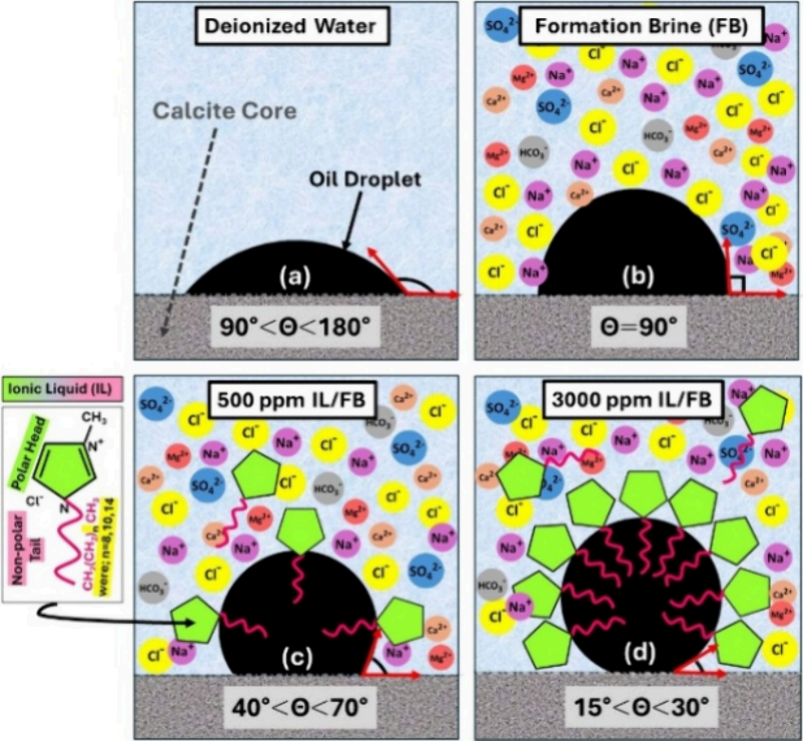
Schematic Mechanism and Contact Angle for SA Oil Droplet in the Presence of a) Deionized Water, b) Formation Brine and c) Imidazolium ILs Diluted in Formation Brine at 500 ppm and d) 3000 ppm

However, the efficiency of C_12_mimBF_4_ in formation brine to modify the wettability to water-wet regimes was lower than the other ILs at 500 and 3000 ppm. Table 4 shows a very low pH (pH < 2) of C_12_mimBF_4_ diluted in formation brine above a concentration of 500 ppm. The stability and surface activity of ILs may be enormously influenced by the pH of the solution. At 500 ppm, C_12_mimBF_4_ in seawater (pH ≈ 6.3) reduced the contact angle to 56.43°, which was comparable to C_12_mimCl (59.57°). This indicates effective wettability alteration under mildly acidic to near-neutral conditions. However, in formation brine at the same concentration of C_12_mimBF_4_, the pH dropped sharply to 2.42. As a result, the contact angle was high (77.83°), reflecting weaker alteration. This reduced efficiency is likely due to the protonation of the imidazolium ring under highly acidic conditions, which can diminish the surface-active properties and aggregation behavior of the IL.^54^ This protonation can decrease the efficiency of C_12_mimBF_4_ to modify the reservoir rock’s wettability. In addition, protonation can affect the aggregation behavior of the IL, which is crucial for its ability to adsorb on and modify the rock surface.

As highlighted by Noruzi et al. (2024), surface charge controls whether oil-wet components are retained or displaced.^51^ In this context, the reduced wettability alteration observed with acidic ILs such as C_12_mimBF_4_ in this study may be attributed to unfavorable electrostatic interactions with the positively charged carbonate surface under acidic conditions. Additionally, the influence of C_12_mimBF_4_ on wettability may be further diminished owing to competition with BF_4_^–1^ for adsorption sites on the rock surface caused by the presence of other ions in the formation brine.^55^

To investigate the influence of IL concentration on wettability, contact angles were assessed for imidazolium-based ILs diluted in both seawater and formation brine at a concentration of 3000 ppm (Figure 12). A decrease in the contact angle of aqueous IL droplets was observed as the concentration of the IL increased from 500 to 3000 ppm. This trend indicates that higher concentrations of ILs could enhance the shift toward more water-wet conditions (Scheme 2d), which is consistent with earlier studies.^56^ The contact angle was altered from 94.42° to 28.08°, 25.57°, 22.27° and 20.63° (a reduction of 70.31%, 72.92%, 76.41%, and 78.15%) with the introduction of C_10_mimCl, C_12_mimCl, C_12_mimBF_4_, and C_16_mimBr at a concentration of 3000 ppm in seawater, whereas the contact angle between formation brine and SA oil decreased from 90.61° to 29.53°, 24.66°, 46.70°, and 15.37°, equivalent to a reduction of 67.41%, 72.78%, 48.46%, and an outstanding 83.04% following the addition of the same ILs at 3000 ppm, respectively.

**Figure 12:**
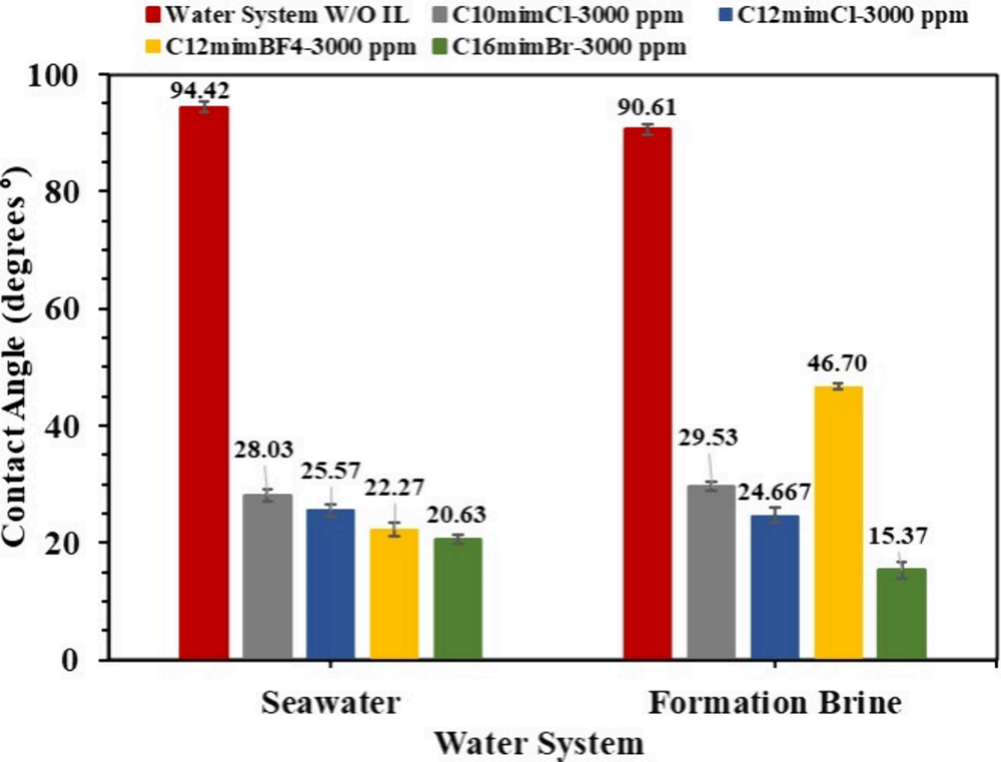
Wettability (contact angle) of the light crude oil and the promising ILs diluted in seawater and formation brine at 3000 ppm.

### 3.6 Adsorption Behavior of Imidazolium ILs on Carbonate Rock

The adsorption behavior of imidazolium-based ILs diluted in formation brine (3000 ppm) was evaluated to understand their interaction with carbonate rocks. Among the tested ILs, C_10_mimCl exhibited the highest adsorption capacity (17.68 mg/g, 23.57%), while C_12_mimBF_4_ showed the lowest (2.71 mg/g, 3.62%) as shown in Figure 13. Adsorption tended to decrease with increasing alkyl chain length beyond C_10_, likely due to steric hindrance and molecular aggregation that limit surface accessibility.^57,58^ The long chain IL C_16_mimBr shows distinctive adsorption capacity of 5.25 mg/g compared to shorter alkyl imidazolium ILs such as C_10_mimCl, and C_12_mimCl. This adsorption behavior makes C_16_mimBr a promising candidate for carbonate EOR schemes. These characteristics mean that sufficient IL remains mobile in the aqueous phase, maximizing IFT reduction and wetting changes without excessive chemical loss to the rock.^59^

**Figure 13:**
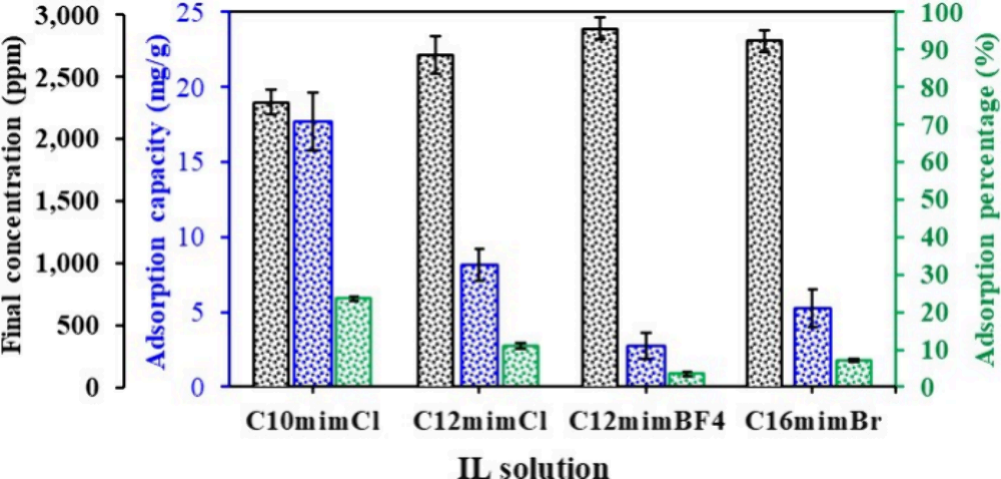
Adsorption behavior of imidazolium-based ILs on carbonate rock.

Additionally, ILs containing Cl^–^ and Br^–^ anions showed stronger interaction with the carbonate surface due to favorable electrostatic interactions, whereas the bulky BF_4_^–^ anion in C_12_mimBF_4_ reduced affinity for mineral surfaces.^60^ While strong IL adsorption onto rocks may suggest surface interaction, high adsorption is generally unfavorable for enhanced oil recovery (EOR). ILs lost to the rock surface cannot participate in key mechanisms such as interfacial tension (IFT) reduction or wettability alteration at the oil–brine interface. Therefore, lower adsorption onto rock surfaces is preferred, as it enhances IL availability in the pore space and reduces chemical loss and operational costs.^61^ Despite its low adsorption, C_12_mimBF_4_ was not the most effective IL overall, likely due to its lower pH as discussed in the wettability section (3.4), which hindered its ability to shift the wettability of carbonate surfaces. Thus, an optimal IL for EOR should achieve moderate adsorption—to retain interfacial activity—while maintaining favorable pH, mobility, and interfacial action in reservoir conditions.

## 4 Conclusions

The potential of imidazolium-based ILs (C_10_mimCl, C_12_mimCl, C_12_mimBF_4_, and C_16_mimBr) as substitutes for traditional surfactants in tertiary oil recovery was investigated. These ILs demonstrated significant IFT reduction, wettability alteration, and thermal stability, offering a novel approach to address global energy demands. This study is the first to examine the combined effects of IL concentration, temperature, and salinity on EOR in Emirati light crude oil, particularly in tight reservoirs. Thermal stability tests (TGA) revealed degradation temperatures between 273.59 and 446.99 °C, with longer alkyl chain ILs (C_12_mimBF_4_ and C_16_mimBr) exhibiting greater stability. Phase behavior analysis at 500 ppm and 90 °C indicated the formation of Winsor Type III emulsions, favorable for EOR, with ILs having longer alkyl chains proving more effective in emulsifying and breaking down accumulated oil. Furthermore, all tested imidazolium ILs at 500 ppm effectively stabilized oil/brine emulsions, with no phase separation observed within 48 h.

IFT reductions were observed across concentrations of 500 to 3000 ppm, attributed to the amphiphilic nature of ILs that weakens oil–water intermolecular forces. The salting-out effect and the presence of divalent ions in formation brine further enhanced IFT reduction, with the lowest IFT recorded at 0.089 mN/m for C_16_mimBr at 110 °C and 3000 ppm. Elevated temperatures enhanced molecular movement, leading to greater IFT reduction and improved efficiency. The ILs also significantly shifted wettability toward more water-wet conditions, with contact angles reduced to 15.37° using C_16_mimBr at 3000 ppm, highlighting the importance of alkyl chain length and concentration in optimizing wettability for EOR. In addition, the adsorption study showed that imidazolium ILs with longer alkyl chains (C_16_mimBr) and bulkier anions (C_12_mimBF_4_) exhibit lower retention down to 2.71 mg/g of carbonate rock, reducing chemical loss and enhancing their availability for EOR mechanisms. The study also underscores the importance of optimizing IL concentration and operational conditions tailored to specific reservoir environments.

This study focused on screening the EOR potential of imidazolium-based ILs through interfacial tension reduction, wettability alteration, adsorption, thermal stability, salinity tolerance, emulsion stability, and microemulsion analysis. Based on these promising results, core-flooding experiments were conducted and will be presented in future research work to evaluate the actual oil recovery performance under reservoir conditions. Therefore, this research provides useful insights for designing more effective and adaptable EOR solutions. It also supports improved sustainability and efficiency in oil recovery. Moreover, the findings are relevant beyond academic interest. They offer practical value for the oil industry, particularly in regions with harsh reservoir conditions such as the UAE.
